# Acknowledgement to Reviewers of *Pathogens* in 2018

**DOI:** 10.3390/pathogens8010005

**Published:** 2019-01-08

**Authors:** Pathogens Editorial Office

**Affiliations:** MDPI, St. Alban-Anlage 66, 4052 Basel, Switzerland

Rigorous peer-review is the corner-stone of high-quality academic publishing. The editorial team greatly appreciates the reviewers who contributed their knowledge and expertise to the journal’s editorial process over the past 12 months. In 2018, a total of 93 papers were published in the journal, with a median time to first decision of 15 days and a median time to publication of 40 days. The editors would like to express their sincere gratitude to the following reviewers for their cooperation and dedication in 2018:

Abraham, Wolf-RainerGupta, KajalPeng, TengAkio, NakaneHairgrove, ThomasPerez-Gracia, Maria-TeresaAllen, DavidHalle, StephanPerez-Morga, DavidAlsford, SamHardwidge, PhilipPetrova, Olga E.Alvarez-Dominguez, CarmenHarmon, Carrie LapairePeyambari, MahtabAmbro, LubosHefferon, Kathleen LauraPortilla, MaribelAnderson, JamesHeinonen-Tanski, HelviPoudel, BarunAndré, Marcos RogérioHenry, Brandon MichaelPrigigallo, Maria IsabellaAnsari, Mohammad AzamHetman, MichalRadcliff, Fiona JaneArmstrong, GlenHill, Kent L.Rather, Irfan AhmadArthur, ArthurHo, HonhingRaval, ManmeetAttardo, Geoffrey M.Horhat, Florin GeorgeReichel, Michael P.Audenaert, KrisHughes, GrantReif, Kathryn E.Azab, WalidHummel, MaryRentsch, DorisBarman, ApurbaHumphry, RogerRoditi, IsabelBaskakov, IliaIijima, NoriakiRodrigues, Celia F.Bélard, SabineIson, Michael G.Rodríguez-López, PedroBeld, JorisJat, Parmjit S.Röltgen, KatharinaBengtsson-Palme, JohanJiang, SizunRomeralo, CarmenBergholz, TeresaJohannsen, EricRonholm, JenniferBerta, GraziellaJohnson, NicholasRoze, Ludmila V.Bessa, LucindaJordao, LuisaRuddraraju, Kasi ViswanatharajuBhave, DevayaniJurak, IgorRusso, AnnapinaBiegalke, BonitaJuszczak, LesławSandle, TimBiernasiuk, AnnaKalsi, MeghaSaraiva, MargaridaBolton, NatalieKamath, DivyaSarkar, MrinalBožović, MijatKaplan, ShaunaSavoie, Jean-MichelBradfute, StevenKato, KeitaScholte, FlorineBrazee, NicholasKeyhani, NematSelim, SamehBreak, TimothyKobayashi, NobumichiShao, QiangBrindley, MelindaKonstantinidis, Theocharis G.Sharma, AnuragBrun, RetoKozieł, EdmundSharma, AtulBruschi, FabrizioKumar, AnilSigusch, Bernd W.Bubici, GiovanniKumar, BinodSimões, Marta FilipaCameron, DanielKumar, GauravSnoeck, Chantal J.Campbell, DavidKumar, RohitashwSoultos, Nikolaos D.Cano, JorgeKumar, SunilSousa, Sílvia A.Chambers, ThomasKunz, WolframStahelin, RobertChang, Hao-XunKuroda, MakotoStamminger, ThomasChang, Kung-ChaoLahiri, AmitStefanon, BrunoChase, ChristopherLambe, TeresaStendahl, OlleCheemarla, NagarjunaLandolt, Gabriele A.Sun, XiaomingChen, Ying-LienLange, DirkSunter, Jack D.Chen, ZiguiLaRock, Christopher N.Surapathrudu, KanakalaChiou, Shyan-SongLetek, MichalSuresh, SubbulakshmiChiu, Tsai-HsinLi, Shao-GangSusi, PetriCoelho, Ana CláudiaLi, YanSymonds, ErinCoelho, CamilaLiang, YuyingCornelison, Christopher T.Cork, SusanLin, XionghaoTai, WanboCorr, SinéadLin, Ying-ChiTajima , MotoshiDaley, PeterLiu, GeorgeTakase, SayakaDandekar, ThomasLiu, JunTeixeira, Miguel C.David, Michael Z.Łobacz, AdrianaTempera, GiannaDe Lillo, EnricoLøbner-Olesen, AndersTempera, ItaloDe Re, ValliLukashevich, Igor S.Terzi, ValeriaDebyser, ZegerLupien, AndréanneTharmalingam, NagendranDeng, XufangLuthra, AmitTiwari, Rakesh K.Desselberger, UlrichLuthra, PriyaTopalidou, EleniDiack, Abigail B.Luvisi, AndreaToranzo, Alicia E.Didonna, AlessandroMacdonald, AndrewToscani, AnitaDíez-Aguilar, MaríaMagiorkinis, EmmanouilTrivedi, PankajDillman, AdlerManet, EvelyneTrobaugh, DerekDkhar, Hedwin KitdorlangMarlicz, WojciechTuplin, AndrewDobrovolny, Hana M.Martínez, Luis CarlosUpadhyay, AbhinavDosoky, Noura S.Merda, DéborahUwiera, RichardDou, ZhichengMetz, Stefan W.Van Bocxlaer, KatrienDrlica, KarlMiki, YasuhiroViducic, DarijaEast, MalcolmModhiran, NaphakVigneron, AurelienEl-Hage, NaziraMonroy, FernandoVillani, AlessandraElShaer, AmrMontagna, Maria TeresaVlasova, Anastasia N.Evermann, JimMoore, Matthew D.Vylkova, SlavenaEwan, PlantMorris, MhairiWalter, MonikaFan, LiMoutelikova, RomanaWang, ZheFan, WeiMünz, ChristianWay, Sing SingFarrow, Mark A.Murata, TakayukiWeele, Pascal Van DerFranz, EelcoMusch, MarkWernike, KerstinFrentiu, FrancescaNaik, SurabhiWertz, Philip W.Fusco, VincenzinaNevárez-Moorillón, Guadalupe VirginiaWhitehurst, ChristopherFutoma-Kołoch, BożenaNiemirowicz, Gabriela T.Wilkinson, BrianGarcía Castañeda, Javier EduardoNissapatorn, VeeranootWillenborg, MarenGarcia-Diaz, JuliaNobbs, AngelaWojtyczka, RobertGarcía-Díez, JuanO’Dea, Mark A.Wullt, BjornGhosh, SouvikO’Connor, ChristineXing, JunjiGogulamudi, Venkateswara ReddyOh, Seung-YoonYang, TuoGold, BenOkino, Cintia HiromiYli-Mattila, TapaniGolos, Thaddeus G.Ortiz-Urquiza, AlmudenaZampese, EnricoGonella, ElenaOsorio, Elvia YanethZhang, FanGong, LiminPaeshuyse, JanZhang, Ling-juanGoodhead, IanPan, YangangZhang, RuiyongGoodman, LauraParadkar, PrasadZhong, GuangmingGoyal, RajniPark, Hyun-WooZhou, BangjunGradoni, LuigiPassler, ThomasZhu, LuchangGuldimann, ClaudiaPeng, Chen


